# Inflammatory myofibroblastic tumor of the right renal pelvis: A case report

**DOI:** 10.1016/j.eucr.2025.102969

**Published:** 2025-01-29

**Authors:** Xiaorui Zhu, Xueli Dong, Hongzhou Sheng, Renbin Deng, Xianzhong Duan

**Affiliations:** Department of Urology, The Second People's Hospital of Baoshan, No.13 Zhengyang South Road, Longyang District, Baoshan, Yunnan, China

**Keywords:** Inflammatory myofibroblastic tumor, Renal pelvis, Clinicopathologic characteristics, Treatment

## Abstract

Inflammatory myofibroblastic tumor(IMT) is an uncommon soft tissue neoplasm rarely reported in the urinary tract. A 54-year-old male presented to our institution with low back and abdominal pain, hematuria, and lower urinary tract symptoms for 2 months. We performed abdominal contrast-enhanced computed tomography (CT) and magnetic resonance imaging (MRI), which showed a mass in the right renal pelvis-inferior calyx. Then, we performed the laparoscopic radical nephroureterectomy.

## 1 Introduction

IMT is a rare mesenchymal tumor, commonly found in the lungs and rarely in the urinary tract, especially the renal pelvis.^1^ Initially considered a benign inflammatory process, IMT is now recognized for its potential malignancy due to chromosomal abnormalities in the p21-23 region of chromosome 2.^2^ Herein, we report a case of a patient with right renal pelvis IMT who underwent laparoscopic radical nephroureterectomy.

## 2 Case report

**Fig. 1:**
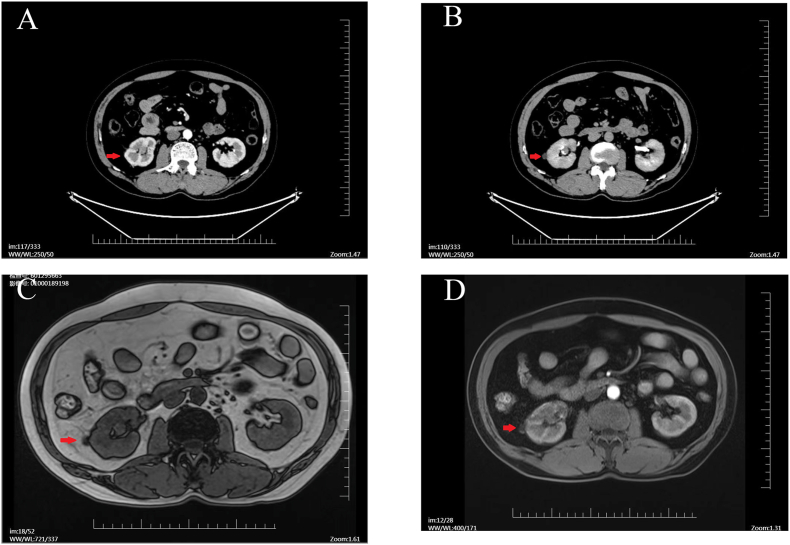
**Pre-operative imaging data**. A–B: CT images. C–D: MRI images.

In April 2023, a 54-year-old male presented to our institution with low back and abdominal pain, hematuria, and lower urinary tract symptoms for 2 months. He denied malignancy, abdominal trauma, and a history of smoking, and a routine physical examination revealed no significant abnormalities. His initial urinalysis indicated elevated leukocytes and significant occult blood, though other laboratory values were normal. To clarify the source of hematuria and pain, we performed abdominal contrast-enhanced computed tomography (CT), which showed a mass of approximately 1.1 × 1.2 × 2.2 cm in the right renal pelvis-inferior calyx, with heterogeneous enhancement in the arterial phase, slightly diminished in the venous phase, and the renal pelvis showing a filling defect in the delayed phase(Fig. 1A and B). Considering a high probability of a malignant tumor of the renal pelvis, we performed a urine cytology, and the results were negative. Magnetic resonance imaging (MRI) was then performed, and the results indicated a soft-tissue mass in the right renal pelvis-inferior calyx with irregular morphology, weak enhancement, and diffusion-limited in the diffusion-weighted imaging (DWI) (Fig. 1C and D), which was consistent with the CT diagnosis.

**Fig. 2:**
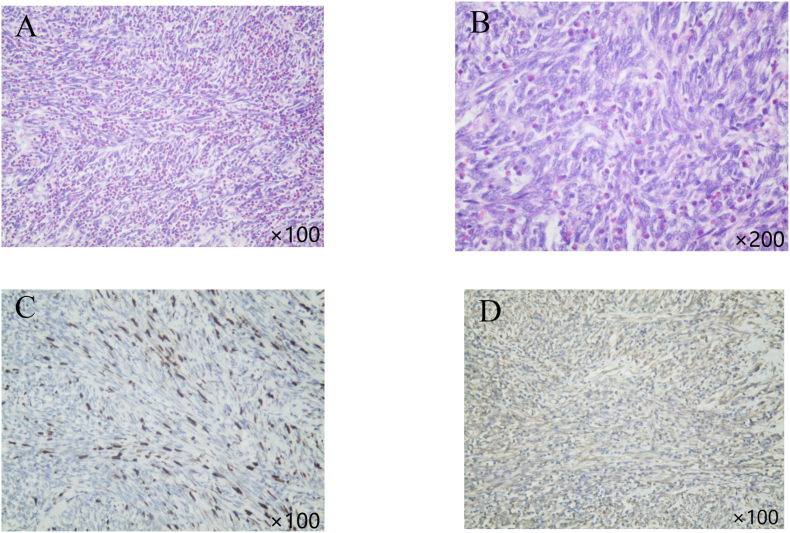
**Immunohistochemistry**. A–B: Immunohistochemistry for Ki-67. C–D: Immunohistochemistry for Vimentin.

The patient ultimately underwent laparoscopic radical nephroureterectomy. The postoperative histopathological examination revealed a generalized distribution of spindle cells with various types of inflammatory cell infiltration including lymphocytes and plasma cells, capillary proliferation, and local necrosis (Fig. 2A and B). Immunohistochemistry was positive for smooth muscle actin (SMA), Ki-67 (20 %), CD99, and vimentin, and negative for activin receptor-like kinase (ALK), STAT6, and CD117 (Fig. 2C and D). The patient's postoperative recovery was uneventful, with no recurrence or metastasis noted. The renal function of the postoperative patient rose from 73.0 μmol/L to 148.3 μmol/L. There was no further increase in the following months.

## 3 Discussion and conclusion

The etiology and pathogenesis of IMT remain elusive at present which may be attributed to chronic inflammation, surgery, trauma, and chromosomal aberrations.^1^ Renal IMT lacks characteristic features in clinical manifestations and imaging exams, with approximately 38%–54 % of patients presenting with low back pain or abdominal pain and 28%–29 % presenting with gross or microscopic hematuria,^3^ due to the presence of extensive fibrous tissue components in the solid mass of IMT, it shows mild enhancement in the arterial phase on enhanced CT scan and low signal intensity in the T2 phase on MRI examination.^4^ Patients are therefore difficult to discriminate preoperatively from renal pelvic malignancies, a fact that often leads to over-surgical treatment. Although there is no clear standard for the treatment of renal IMT, radical surgical resection is primarily performed.^5^ In our case, the middle-aged male displayed symptoms and imaging akin to renal pelvis cancer. Following a laparoscopic radical nephroureterectomy, he experienced no severe complications or signs of recurrence, suggesting that surgery is an effective IMT treatment. However, its long-term prognosis requires further study.

## CRediT authorship contribution statement

**Xiaorui Zhu:** Writing – original draft, Methodology, Investigation, Funding acquisition, Formal analysis, Data curation, Conceptualization. **Xueli Dong:** Validation, Software, Project administration, Methodology. **Hongzhou Sheng:** Validation, Supervision, Methodology, Investigation, Conceptualization. **Renbin Deng:** Writing – review & editing, Software. **Xianzhong Duan:** Writing – review & editing, Supervision, Project administration, Investigation.

## Consent to participate

Informed written consent was obtained from patients to participate in this study.

## Ethics approval

Ethics approval was granted from The Second People's Hospital of Baoshan.

## Consent for publication

All authors have agreed to publish this paper.

## Funding

This study is funded by the 10.13039/501100007846Yunnan Provincial Department of Education Science Research Fund Project (grant no. 2022J1574).

## Declaration of competing interest

None.
